# Skin epidermal keratinocyte p53 induces food uptake upon UV exposure

**DOI:** 10.3389/fnbeh.2023.1281274

**Published:** 2023-12-12

**Authors:** Shivang Parikh, Roma Parikh, Marco Harari, Aron Weller, Lior Bikovski, Carmit Levy

**Affiliations:** ^1^Department of Human Genetics and Biochemistry, Faculty of Medicine, Tel Aviv University, Tel Aviv, Israel; ^2^Dead Sea and Arava Science Center, Masada DMZ Medical Center, Ein Bokek, Israel; ^3^Faculty of Medicine, Hebrew University, Jerusalem, Israel; ^4^Department of Psychology and the Gonda Brain Research Center, Bar-Ilan University, Ramat Gan, Israel; ^5^The Myers Neuro-Behavioral Core Facility, Tel Aviv University, Tel Aviv, Israel; ^6^School of Behavioral Sciences, Netanya Academic College, Netanya, Israel

**Keywords:** UVB exposure, epidermal keratinocytes, p53 activation, anxiety-related behaviors, food intake motivation, conditioned-place preference, mice

## Abstract

**Introduction:**

The first cells affected by UVB exposure are epidermal keratinocytes, and p53, the genome guardian, is activated in these cells when skin is exposed to UVB. UVB exposure induces appetite, but it remains unclear whether p53 in epidermal keratinocytes plays a role in this appetite stimulation.

**Results:**

Here we found that food intake was increased following chronic daily UVB exposure in a manner that depends on p53 expression in epidermal keratinocytes. *p53* conditional knockout in epidermal keratinocytes reduced food intake in mice upon UVB exposure.

**Methods:**

To investigate the effects of p53 activation following UVB exposure, mice behavior was assessed using the staircase, open-field, elevated-plus maze, and conditioned-place preference tests. In addition to effects on appetite, loss of p53 resulted in anxiety-related behaviors with no effect on activity level.

**Discussion:**

Since skin p53 induces production of β-endorphin, our data suggest that UVB-mediated activation of p53 results in an increase in β-endorphin levels which in turn influences appetite. Our study positions UVB as a central environmental factor in systemic behavior and has implications for the treatment of eating and anxiety-related disorders.

## 1 Introduction

UV was recognized as a carcinogen in 1928, sparking a behavioral trend of minimizing sun exposure (Findlay, 1979; Chang et al., 2014). Subsequent epidemiological studies indicated that the role of UV in human health is complex as UV exposure can extend life expectancy due to protection against cardiovascular disease and other causes of mortality (Lindqvist et al., 2014, 2016a,b) and can reduce the risk of endometrial (Epstein et al., 2009) and colon (Garland and Garland, 1980) cancers. Moreover, sun exposure causes an increase in liver metabolism, especially of triglycerides, protecting the organ from hepatocellular lipotoxicity (Ferguson et al., 2019) and metabolic disease (Ferguson et al., 2019).

The health benefits of solar radiation have been attributed to vitamin D (Wacker and Holick, 2013), but this assumption was called into question by analysis of two large-scale clinical trials that demonstrated that vitamin D alone is not associated with reduced risk of cardiovascular disease, all-cause mortality, or invasive cancer (Manson et al., 2019). A literature survey of over 100 studies also suggested that sun exposure has a beneficial effect on cardiovascular diseases that is not associated with vitamin D (Weller, 2016). UV radiation upregulates local neuroendocrine axes, which can affect not only organ function but also the central nervous system to influence addictive and sexual behaviors (Fell et al., 2014; Parikh et al., 2021). UVB is markedly more efficient than UVA in inducing behavioral changes (Slominski et al., 2018). These findings indicate that at least some of the health benefits of sunlight are independent of vitamin D.

Few studies done by our research group on human subjects and C57BL/6 mice indicate that UVB exposure influences both sexual and food-seeking behavior and that these effects are mediated via the skin (Parikh et al., 2021, 2022, 2023). Skin has three layers, the epidermis, the dermis, and the hypodermis (Agarwal and Krishnamurthy, 2023). We demonstrated that UVB-induced sexual behavior and hormonal changes are mediated by p53 activation in epidermal keratinocytes; p53 activates the hypothalamus-pituitary-gonadal axis, which induces sexual behavior and ovarian pro-fertility (Parikh et al., 2021). Further, we discovered that daily low-level UVB exposure enhances the food-seeking behavior of male mice but not of female mice (Parikh et al., 2022). The appetite enhancement caused by UVB exposure results from elevation of secretion of ghrelin from skin adipocytes in males; in females, estrogen inhibits p53 activity in adipocytes, thus blocking elevation of ghrelin levels (Parikh et al., 2022), demonstrating sex dependent response to UVB radiation.

Another effect of UVB exposure is p53 induction in skin keratinocytes (Fell et al., 2014). p53 induces production of β-endorphin, which is an opioid cleaved from pro-opiomelanocortin (Zhou et al., 1993; Sprouse-Blum et al., 2010), that induces addiction-like behavior and euphoria and that has rewarding and reinforcing properties (Roth-Deri et al., 2008; Veening and Barendregt, 2015). In addition, there is evidence that β-endorphin is involved in reward pathways (Dum, 1983) that override satiation signals of food consumption, suggesting that β-endorphin plays a role in motivational behavior (Alonso-Alonso et al., 2015). Mice consume food beyond that necessary for growth or homeostasis when the reward pathways are triggered (Berridge, 1996; Egecioglu et al., 2010; Berthoud et al., 2011). Thus, β-endorphin is associated with increased feeding behavior (Dutia et al., 2012; Nogueiras et al., 2012). Finally, keratinocyte epidermal p53 induces skin β-endorphin expression upon UVB exposure (Fell et al., 2014), however, it was never shown whether skin p53 induced upon UV has effect on the β-endorphin related feeding behavior.

In this study, we investigated the p53-dependent changes in feeding behavior of C57BL/6 J mice induced by UVB exposure. Data from behavioral models, including the staircase, the open-field, the elevated-plus maze, and the conditioned-place preference (CPP) tests, demonstrated that UVB affects eating as well as anxiety-like behavior in an epidermal p53-dependent manner without changing total activity levels. These results were corroborated using mice with *p53* conditional knockout in keratinocytes. Our data indicate that β-endorphin production in skin epidermis induced by UVB enhances appetite.

## 2 Results

### 2.1 UVB exposure increases food intake

We previously used a chronic UVB exposure mouse model by shaving the dorsal hair and exposing male mice to either mock-irradiation (control) or UVB at a dose of 50 mJ/cm^2^ daily for 10 weeks (Parikh et al., 2022). This dose is approximately equal to 25–30 min of midday ambient solar exposure in Florida on a sunny day for a person with the Fitzpatrick phototype 2–3 (D’Orazio et al., 2006; Fell et al., 2014). This dose did not cause any major skin damage as shown previously (Parikh et al., 2022). Feeding behavior is a reflection of underlying physiological and molecular processes and responses to the environment (Parikh et al., 2022), stress (Francois et al., 2022), disease (Adebakin et al., 2012), transgene expression (Newmyer et al., 2019), or drug or hormone treatment (Ghule et al., 2020; Yoon et al., 2020). To investigate feeding behavior following UVB exposure, by feeding the animals with sucrose pellets, we perform an initial calibrating experiment which demonstrate a significant higher food consumption was in the UVB-treatment group compared to the mock irradiated (control group) (Supplementary Figures S1A–C) as per the statistical *t*-test *p* value 0.0003, *t*-statistics (*t* = 3.696, df = 142), and *F*-statistics (*F* = 1.218, DFn = 71, DFd = 71). These results indicate that sucrose pellets consumption is selectively increased due to UVB exposure.

**Figure 1:**
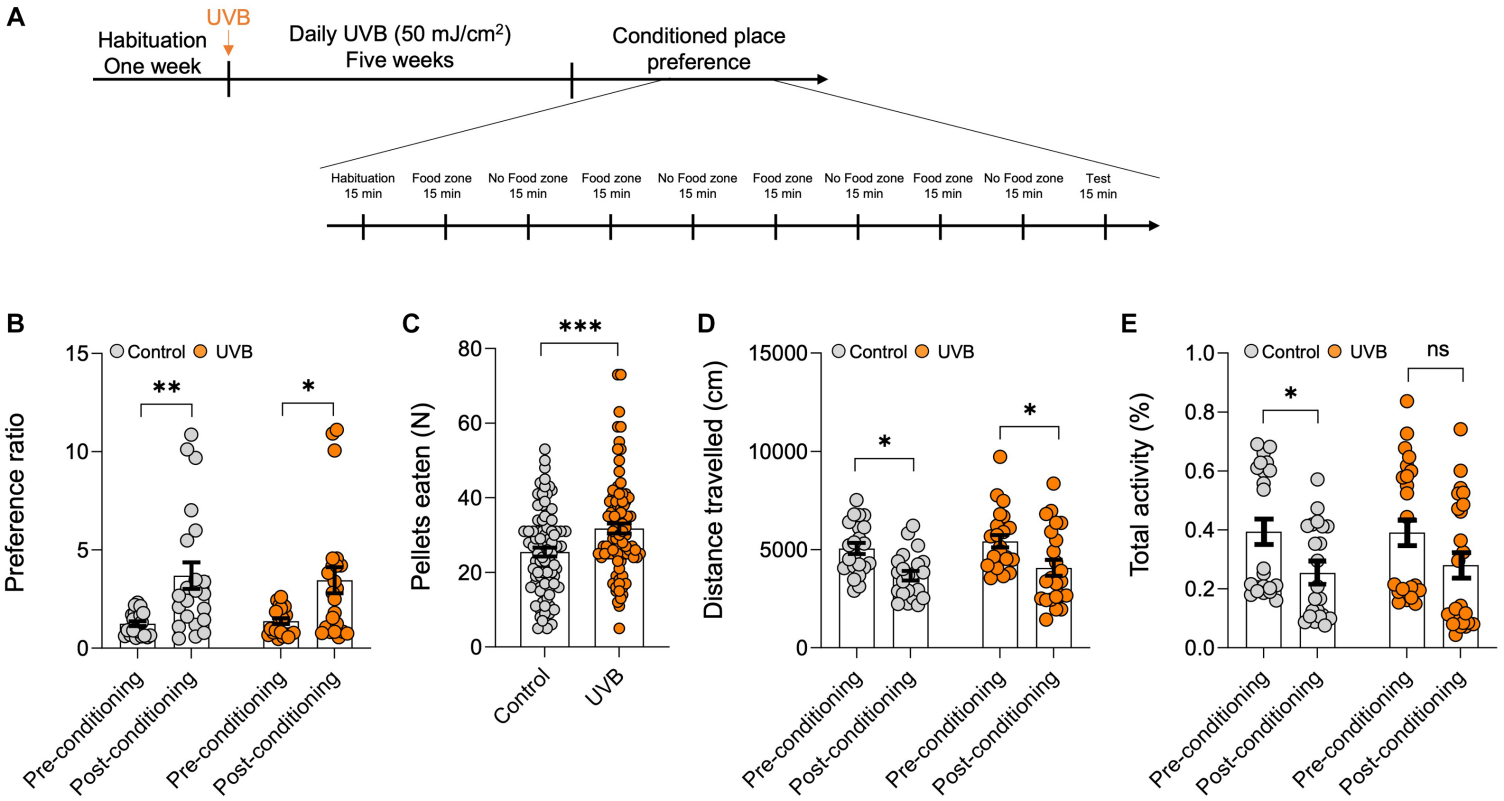
UVB exposure increases food intake. **(A)** Experimental scheme for the open field test. C57BL/6 J male mice were habituated for 1 week followed by daily mock irradiation (control) or 50 mJ/cm^2^ UVB exposure for 5 weeks. At the end of the fifth week, the animals were subjected to the CPP test. The total duration of the CPP test was 9 days. **(B)** Preference ratios, calculated by dividing the cumulative time spent in side A by the cumulative time spent on sides **(A,B)** of the CPP arena, during pre-conditioning (Day 0) and post-conditioning (Day 9) (n ≥ 22 mice per group). Data for individuals are plotted as dots, and means ± SEM are shown as a bar graph. Significance was evaluated by two-way ANOVA for conditioning as a source of variation (***p < 0.0001) and by Tukey’s multiple comparison for pre-conditioning vs. post-conditioning (**p < 0.01, *p < 0.05). Statistical details are provided in Supplementary Table S1. **(C)** Average food pellets consumed in 15-min CPP test session by control and UVB-treated mice on days 2, 4, 6, and 8. Data for individuals are plotted as dots and means ± SEM are shown as a bar graph. Two-tailed unpaired *t*-tests were performed (*p < 0.05). **(D,E**) Average distance traveled (in cm) and **(E)** total activity time (in %) during the CPP test. (*n* ≥ 22 mice per group). Data for individuals are plotted as dots and means ± SEM are shown as a bar graph. Significance was evaluated by two-way ANOVA for conditioning as a source of variation (***p < 0.0001) and Tukey’s multiple comparison for pre-conditioning vs. post-conditioning (*p < 0.05). Statistical details are provided in Supplementary Table S1.

Food intake increases can be associated with homeostatic or hedonic behavior. Hedonic consumption is motivation rather than metabolism dependent (Noye Tuplin et al., 2019). To further determine whether the increase in appetite (for reward food) resulting from UVB treatment was a homeostatic or a hedonic behavior, we used the CPP test, which has been used to measure the motivational effects of objects or experiences (Tzschentke, 2007). Our experiment included control and UVB-exposed mice. Mice were habituated for 1 week, were mock-irradiated or UVB irradiated daily for 5 weeks, and were then subjected to the CPP regime for 10 days (Figure 1A). For the CPP, mice were habituated on Day 1, on Days 2–9, mice were exposed on alternate days to food (sucrose pellets) or no reward food zones, and Day 10 was the test day. We found no difference in the preference ratio for a particular side of the arena during pre-conditioning versus post-conditioning between control and UVB-treated mice (Figure 1B) as per the Two-way ANOVA (*p* value <0.0001), and *F*-statistics [F (DFn, DFd) = *F* (1, 86) = 21.95]. We did find, however, that UVB-treated mice consumed significantly more food during the conditioned stage of the experiment than did control mice (Figure 1C) as per the statistical *t*-test *p* value 0.0006, *t*-statistics (*t* = 3.510, df = 178), and *F*-statistics (*F* = 1.304, DFn = 91, DFd = 87), which is in line with our previous study (Parikh et al., 2022). No differences in distance traveled or total activity were observed between groups (Figures 1D,E), but Two-way ANOVA revealed that the conditioned place preference factor without the UVB factor was significant as for the distance traveled [*p* value <0.0001, and *F*-statistics (F (DFn, DFd) = F (1, 86) = 18.29)] and the total activity [*p* value 0.0055, and *F*-statistics (F (DFn, DFd) = F (1, 86) = 8.109)] (Supplementary Table S1), which ruled out the possibility that higher motor activity increased feeding behavior. Taken together, our CPP data indicate that chronic daily UVB exposure does not induce activity-related effects on food consumption but rather increases food-seeking behavior in mice.

### 2.2 p53 Knockout in epidermal keratinocytes have significant but modest effects on UVB-induced food intake

**Figure 2:**
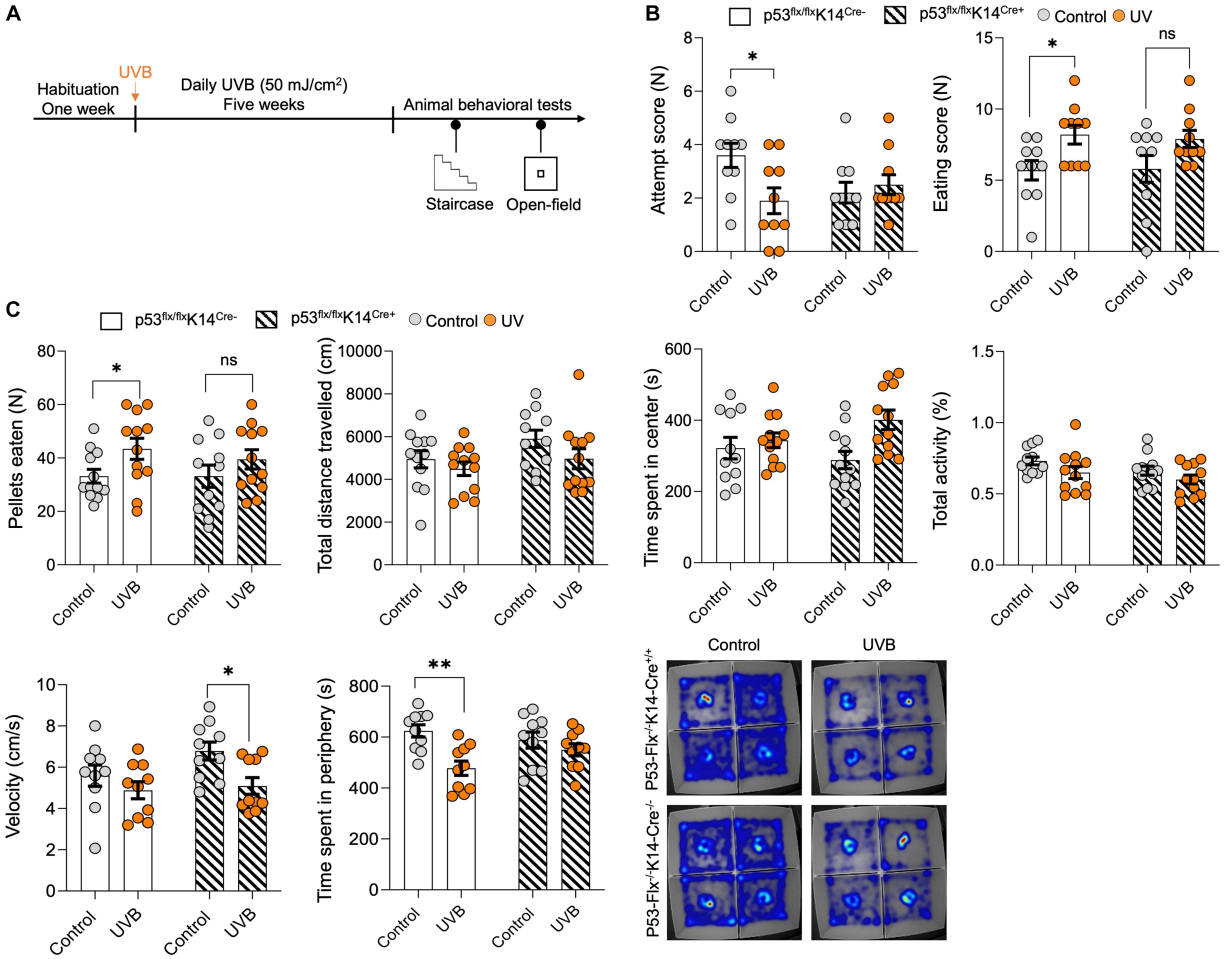
*p53* knockout in epidermal keratinocytes have significant but modest effects on UVB-induced reward food intake. **(A)** Experimental scheme for the open field and staircase test. Wild-type mice and mice with conditional knockout of *p53* in keratinocytes were habituated for 1 week followed by daily mock irradiation or 50 mJ/cm^2^ UVB exposure for 5 weeks. At the end of the fifth week, the animals were deprived of food for 22 h and then subjected to the staircase and open field tests. **(B)** Number of attempts (left) and the eating scores (right) for the control and UVB-treated male mice during the staircase test. (*n* ≥ 10 mice per group). Data for individuals are plotted as dots and means ± SEM are shown as a bar graph. Significance was determined by two-way ANOVA analysis with student’s *t*-test. Details are given in Supplementary Table S2. **(C)** Food pellets consumed, total distance traveled (CM), time spent in center (s), total activity (%), velocity (cm/s), and time spent in periphery (s) for the control and UVB-treated male mice during the open-field test (*n* ≥ 11 mice per group). Data for individuals are plotted as dots and means ± SEM are shown as a bar graph. Significance was determined by two-way ANOVA analysis with Tukey’s multiple comparison (for total distance traveled, time spent in center, total activity, velocity, and time spent in periphery) and student’s *t*-test (for the food pellets consumed). Details are given in Supplementary Table S2. Bottom: Representative heat maps for a mouse from each group. *Represents *p*-value < 0.05.

To test whether the UVB-induced behavior changes depend on p53 activation in keratinocytes, we generated a conditional *p53* knockout mouse model (Parikh et al., 2021) in which p53 deletion induced specifically in keratinocytes (p53^flox/flox^K14-Cre^+/+^); wild-type littermates (p53^flox/flox^K14-Cre^−/−^) were used as controls. These mice were exposure to UVB and subjected to the staircase test and open-field tests (Figure 2A). In the staircase test we found significantly greater food pellets consumption [*p* value = 0.0172, *t*-statistics (*t* = 2.624, df = 18), and *F*-statistics (*F* = 1.063, DFn = 9, DFd = 9)] with significantly less attempts of pellets uptake in the UVB treated wild-type mice (Figure 2B). Although less attempts were made by the wild-type mice, these were successful attempts, suggesting that mice spent more time in procuring the food. No significant increase in food consumption following UVB was observed in the p53 cKO animals as shown in Figure 2B [*p* value = 0.0766, *t*-statistics *t* = 1.878, df = 18), and *F*-statistics (*F* = 2.419, DFn = 9, DFd = 9)]. Next, to understand the food behavior in a challenging setting (bright light and open space) we implied the use of the open-field assay as previously done by us (Parikh et al., 2022). Only wild-type mice showed significant induction in the food consumption upon UVB treatment [*p* value 0.0414, *t*-statistics (*t* = 2.617, df = 22), and *F*-statistics (*F* = 2.351, DFn = 11, DFd = 11)] and not the cKO UVB treated mice (*p* value 0.3726, *t*-statistics (*t* = 0.9120, df = 20), and *F*-statistics (*F* = 1.264, DFn = 10, DFd = 10)). Our data strongly suggest that total activity [*p* value = 0.0416, and *F*-statistics (F (DFn, DFd) = *F* (1, 43) = 4.414)], time spent in center [*p* value = 0.0119, and *F*-statistics (F (DFn, DFd) = F (1, 43) = 6.906)], and velocity [*p* value = 0.0102, and *F*-statistics (F (DFn, DFd) = *F* (1, 36) = 7.345)] was significant in UVB mice and the velocity is significantly reduced in the p53-cKO animals UVB animals compared to mock (*p* value = 0.049) (Supplementary Table S2), indicating that there might be a modest decrease in the velocity of UVB-treated animals in open-field test when the p53 is knocked-out in keratinocytes. This compliments the food pellets consumed data, that despite of spending more time in the center zone where food is kept, the p53-cKO UVB treated mice eat less (Figure 2C). Additionally, the time spent in the peripheral areas of the open-field (non-food zone) where the UVB factor is significant [*p* value = 0.0016, and *F*-statistics (F (DFn, DFd) = F (1, 36) = 11.61] and the UVB wild-type mice spent significantly less time in periphery compared to mock wild-type mice (*p* value = 0.0026), suggesting that they spent more time in the center and ate more food pellets (Figure 2C). The detailed Two-way ANONA analysis with *t*-statistics, *F*-statistics, and Tukey’s multiple comparison tests appears in Supplementary Table S2. Overall, our data shows the complex yet strong interplay of the feeding and anxiety behaviors considering the UVB and the p53-cKO as two different variables. Both behavioral assays show the same trend of significant but modest effects of the UVB in wild-type mice and the effect is abrogated in the cKO UVB treated mice.

### 2.3 p53 Knockout in epidermal keratinocytes induces anxiety-related behavior

**Figure 3:**
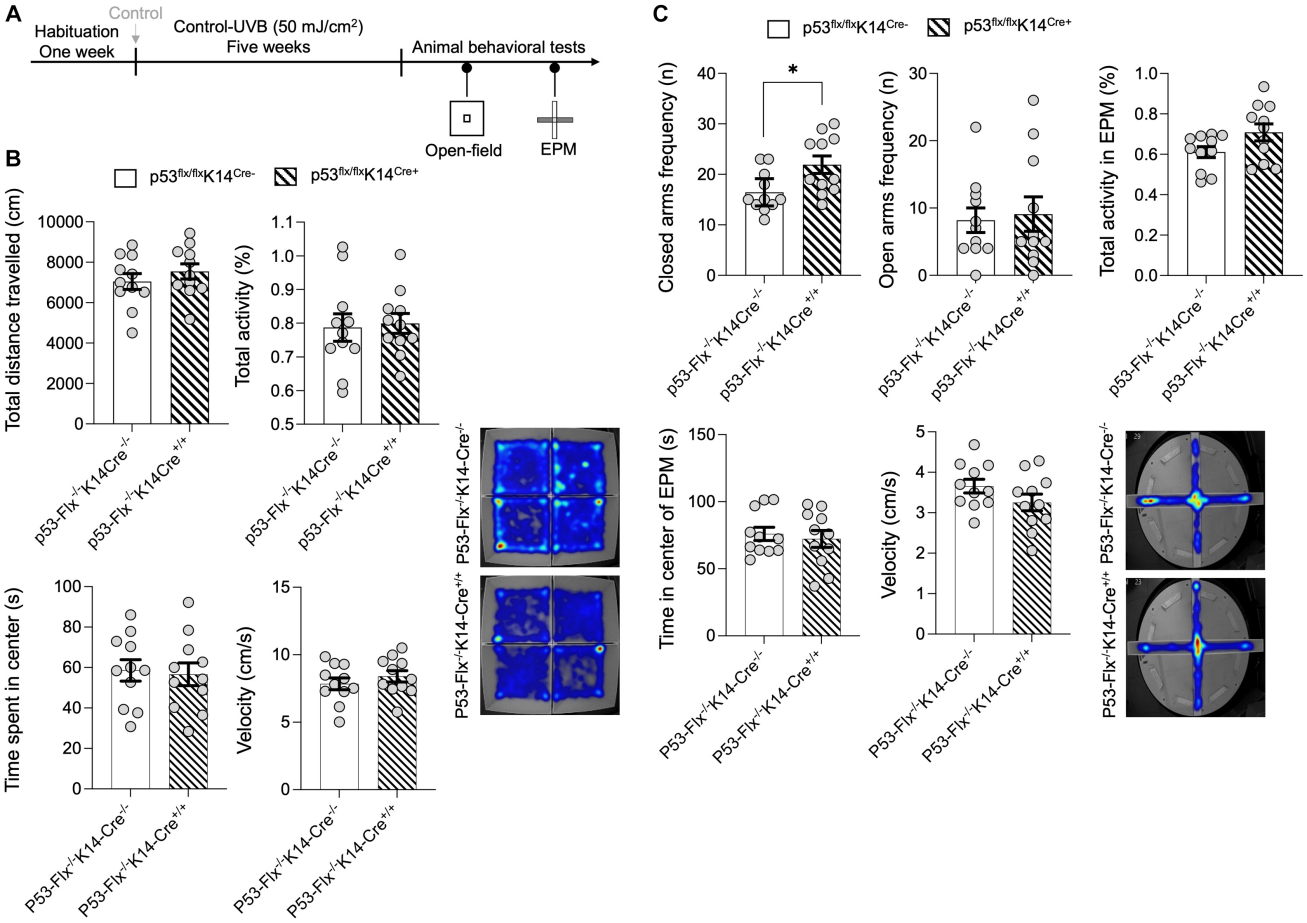
p53 knockout in skin epidermal keratinocytes induces anxiety-related behavior. **(A)** Experimental scheme for the open field test. Wild-type mice and *p53*-knockout mice were habituated for 1 week followed by control^−^UVB exposure for five weeks. At the end of the fifth week, the animals were starved for 22 h and then subjected to the open field test and elevated-plus maze test. **(B)** Total distance traveled (CM), total activity (%), time spent in the center (S), and the velocity (cm/s) in the arena were determined (*n* = 11 mice per group). Data for individuals are plotted as dots and means ± SEM are shown as a bar graph. Details of the statistical analyses are given in Supplementary Table S3. Bottom: Representative heat maps for males (left) and females (right) from each group. **(C)** Total frequency/number of visits in closed and open arms (n), activity, time spent in center (s), and velocity (cm/s) during the elevated-plus maze test. Determined (*n* = 11 mice per group). Data for individuals are plotted as dots and means ± SEM are shown as a bar graph. Details of the statistical analyses are given in Supplementary Table S3. Bottom: Representative heat maps for males from each group. *Represents *p*-value < 0.05.

To further explore the effects of loss of p53 in keratinocytes, we subjected the mice to the open-field test without food and measured total distance traveled in the arena and total activity (Figure 3A). No significant differences between wild-type and mice lacking p53 in keratinocytes were detected (Figure 3B) as per the statistical *t*-test analysis for the distance traveled (*p* value 0.3646, *t*-statistics (*t* = 0.9278, df = 20), and *F*-statistics (*F* = 1.094, DFn = 10, DFd = 10)) and the total activity (*p* value 0.811, *t*-statistics (*t* = 0.2423, df = 20), and *F*-statistics (*F* = 1.919, DFn = 10, DFd = 10)), time spent in the center (*p* value = 0.8025, *t*-statistics (*t* = 0.2534, df = 20), and *F*-statistics (*F* = 1.130, DFn = 10, DFd = 10)), and velocity (*p* value = 0.3636, *t*-statistics (*t* = 0.9297, df = 20), and *F*-statistics (*F* = 1.077, DFn = 10, DFd = 10)). Since skin β-endorphin influences anxiety (Fell et al., 2014) and *p53* knockout results in elevated anxiety- and depression-like behaviors (Ruan et al., 2015), we hypothesized that lack of p53 in the keratinocytes might increase anxiety-like behavior. To test this, we subjected the *p53*-knockout and wild-type mice to the elevated-plus maze test (Parikh et al., 2022) as shown in Figure 3A. We evaluated the number of times/visits (frequency) to the closed and open arms and total activity. A per the statistical *t*-test mice that lack p53 entered the closed arms of the maze more frequently than did wild-type mice (*p* value 0.0177, *t*-statistics (*t* = 2.585, df = 20), and *F*-statistics (*F* = 2.046, DFn = 10, DFd = 10)), although frequency of visits to open arms (*p* value 0.7756, *t*-statistics (*t* = 0.2890, df = 20), and *F*-statistics (*F* = 1.961, DFn = 10, DFd = 10)) and total activity (*p* value 0.0658, *t*-statistics (*t* = 1.946, df = 20), and *F*-statistics (*F* = 2.422, DFn = 10, DFd = 10)) did not differ (Figure 3C). Additionally, there was no significant differences in both velocity (*p* value = 0.1433, *t*-statistics (*t* = 1.524, df = 20), and *F*-statistics (*F* = 1.490, DFn = 10, DFd = 10)) or center time spent (*p* value = 0.6405, *t*-statistics (*t* = 0.4742, df = 20), and *F*-statistics (*F* = 1.619, DFn = 10, DFd = 10)) in the EPM as per the *t*-test. We added a sentence in the result section as shown in new Figure C and the details of the statistics are in Supplementary Table S3. This suggests that the anti-anxiety effect of skin β-endorphin is compromised when p53 is not expressed in keratinocytes.

## 3 Discussion

In this study, we evaluated the effect of daily UVB radiation (50 mJ/cm^2^), mimicking the effects of 20–30 min of exposure to mid-day sun (D’Orazio et al., 2006; Fell et al., 2014), on food intake in mice. UVB exposure increased food consumption in a manner dependent on p53 expressed in epidermal keratinocytes. We propose that p53 is a central regulator of food intake, regulating inhibition mediated by melanocyte-stimulating hormone (MSH), leptin, and insulin and food intake enhancement induced by ghrelin and β-endorphin. MSH is produced from the precursor proopiomelanocortin (POMC) (Akil and Watson, 1980) and is released by POMC neurons, inhibiting food intake inhibition and increasing energy expenditure (Toda et al., 2017). POMC neurons also respond to leptin, an adipocyte-derived satiety hormone that blocks food intake (Balthasar et al., 2004). p53 and leptin function in a negative regulatory loop (Yanagi et al., 2018), suggesting that p53 might enhance food intake by blocking leptin activity, a hypothesis that requires further studies. There is also a negative regulatory loop involving p53 and insulin. p53 represses expression of the insulin receptor (Webster et al., 1996), and insulin downregulates p53 levels through signaling mediated by Akt and MDM2 (Abraham and O’Neill, 2014). We demonstrated that when β-endorphin was blocked in the UVB-treated mice using naltrexone there was a significant reduction in the levels of food-consumed in the UVB-treated naltrexone injected animals but still significantly higher compared to UVB-treated vehicle/naltrexone alone (Parikh et al., 2022). The effect was ghrelin-dependent but we cannot exclude the possibility of an overlap in the effects of feeding behavior of beta-endorphin from keratinocytes and ghrelin from adipocytes which might indicate that at peripheral levels it will be difficult to dissect the effect, but the effects would also coincide in the CNS where the reward pathway and ghrelin would coincide. Further, both the opioid and ghrelin axes are modulated by UVB exposure, and both affect feeding behavior. In conclusion, the literature together with our studies suggest that p53 is a central regulator of food intake and maintains the balance between enhancement of food intake enhancement (by ghrelin, or β-endorphin) or inhibition (by leptin, insulin, ⍺-MSH).

p53 stimulates appetite through effects on ghrelin (Chen et al., 2017), which activates the arcuate AgRP/NPY orexigenic neurons to enhance food intake (Porteiro et al., 2013; Quiñones et al., 2018). UVB increases secretion of β-endorphin by keratinocytes, a process also regulated by p53 (Fell et al., 2014). The reward system is activated by β-endorphin (Ratner et al., 2012; Pilozzi et al., 2020), β-endorphin-deficient mice are less motivated to press a lever for reward food (Hayward and Low, 2007), and β-endorphin increases the addictive effects of drugs such as alcohol and cocaine (Roth-Deri et al., 2008). Rather than having inherent rewarding properties, β-endorphin enhances existing properties of a stimuli. The magnitude of the behavioral effect of ghrelin is too robust as seen from our previous study (Parikh et al., 2022) which might dominate the modest behavioral effects in our p53-K14 cKO mouse model for staircase and open-field tests. This can be the limitation of this mouse model as UVB is a common inducer of p53 in the skin for ghrelin and β-endorphin. UVB irradiation had modest effects on the reward food intake in mice lacking p53 in keratinocytes compared to mock-irradiated controls in both the staircase and open-field tests. Thus, p53 expressed in keratinocytes appears to mediate the food increase that follows UVB exposure in a significant but modest manner.

β-endorphin has also moderating effects on stress response (Merenlender-Wagner et al., 2009). To assess the involvement of stress in our results, we studied mice that do not express p53 in keratinocytes and therefore do not secrete β-endorphin in response to UVB. When examined in stress inducing environments of the open-field test and the elevated-plus maze test, mice lacking p53 did not consume more reward pellets in response to UVB exposure. In addition, they did not exhibit increased activity in either test. It may be that β-endorphin enhances explorative behavior under stress conditions but not when visiting a low-stress environment, such as a well habituated chamber of a CPP box. Results reported by Vaanholt et al. support this hypothesis. In their study when confronted with a dominant mouse displaying aggression, mice lacking β-endorphin exhibited elevated stress responses, as indicated by increased corticosterone levels, and demonstrated higher aggressive response. These findings imply that β-endorphin modulates the stress response to facilitate adaptive behavioral reactions to stressors (Vaanholt et al., 2003).

In this work, only male mice were used in the experiment, and it is therefore interesting to see in the future how females would react following p53 cKO in skin keratinocytes. The proportion of the sex hormones (testosterone and or estrogen) varies during different life stages in males and females, and secondary sexual characteristics are also reflected in behavior. For example: (1) In gliomagenesis there are sex differences in the effects of p53 mutations occurring more frequently in males, associated with worse survival (Haupt and Haupt, 2021); (2) Men have significantly greater burden of missense mutations in melanoma compared to women (Gupta et al., 2015); (3) Rodent studies have shown the sexual dimorphism in the behavioral studies like open-field and EPM where female rats showed less anxiety-like behavior (Knight et al., 2012), indicating a baseline sex differences in both anxiety-like behaviors and the role of p53. We discovered that only male mice increased food-seeking behavior upon solar-UVB exposure, and females were protected from this effect because of their high-estrogen levels, using open-field, staircase test, home cages, PhenoTypers methodologies (Parikh et al., 2022). Therefore, we cannot exclude the possibility that the sexual baseline differences and other potential of sex differences may be affected upon p53 epidermal abolishment.

A possible limitation of this study is that our conditional *p53* knockout mouse model may have behaved differently from wild-type controls for reasons downstream to the deletion itself. Collectively, our past and present studies indicate that the different feeding/starvation times following the UVB have different behavioral effects which might involve the underlying mechanisms involving ghrelin, β-endorphin and other yet unknown molecular players. To investigate the additional unknown molecular targets at different feeding/starvation times following the UVB exposure can be an interesting question to explore, and this can be studied with different intensities of the UVB.

## 4 Materials and methods

### 4.1 Mice

All animal experiments were approved by the Tel Aviv University Institutional Animal Care and Use Committee (IACUC) (01–19-003). Mice were housed under a standard 12-h light/dark cycle (lights on at 20:00 pm) at constant temperature (24 ± 1°C) and humidity (50 ± 5%) with *ad libitum* access to food and water at all times except as mentioned. C57BL/6 J (age: 6–8 weeks old) male mice (Envigo) were used for the experiments. For generation and validation of knockout of the conditional knockout of *p53* in keratinocytes, we used the same strategy as we previously reported (Parikh et al., 2021). In brief, the *p53*-floxed mice (a gift from Professor Eli Pikarsky, The Hebrew University of Jerusalem, Israel) were bred with mice in which the *K14* promoter directs expression of Cre recombinase (a gift from Professor Ittai Ben-Porath, The Hebrew University. of Jerusalem, Israel).

### 4.2 UVB exposure

Mice were shaved on the dorsal side in an area of approximately ~60% of the skin using a shaver and remaining hair were removed using depilatory cream (Veet). Mice were exposed individually to 50 mJ/cm^2^ UVB using the standard light setting with a XX-15 stand equipped with 15-W, 302-nm UVB bulbs (Ultraviolet Products) at approximately the same time in the morning daily for 5 weeks. For the mock-irradiated control treatment, the mice underwent the same handling procedure without UVB exposure. The container was cleaned using Virusolve (Amity International) to avoid cross-contamination of odors between mice.

### 4.3 Food consumption tests

Mice were subjected to 1 week of habituation followed by 5 weeks of daily mock irradiation or UVB irradiation and weekly testing of food consumption. In each weekly test, the mice were food-deprived for 3 h in a home cage-like setting with one mouse per cage followed by *ad libitum* access to standard chow (Altromin, Germany, #1318 M) for 1 h and then to peanut-flavored reward food (TestDiet, Richmond, IN, #1811369) for 30 min. The mice were weighed before and after each weekly test. After the test was completed, the mice were housed in their home cages with *ad libitum* food and water. The chow was weighed before and after a mouse was placed in a cage, and chow consumed was calculated. Chow was placed in the cap of the 50 mL tube that was taped inside the cage so that the mouse could not spill the food.

### 4.4 Staircase test

The staircase test was performed as described previously (Parikh et al., 2022) except that mice were starved for 22 h prior to testing. For food deprivation, animals were fed at noon on non-testing days with water *ad libitum*. At the outset of testing, the mice were habituated with the sucrose pellets and then to the test boxes by placing food along the surface of the staircase steps for a 10 min session a day prior to the experimental day, to rule out the effects of neophobia. On the test day the mice were placed in the staircase chamber one mouse at a time. A mirror was used to check the pellets consumed from both the sides. The duration of the test was of 10 min, and pellets eaten, and attempts were manually scored for each animal using media recorder (Noldus Information Technology). Between trials, the staircase chamber was cleaned with Virusolve® + (Amity International).

### 4.5 Open-field test

The open-field was performed as described previously (Parikh et al., 2021) except that mice were starved for 22 h prior to testing. Time spent in the center, total distance traveled in the arena, velocity, time spent in the peripheral region (non-food zone) and activity levels were measured by video tracking using EthoVision. Between each trial, the open-field arena and the tube cap were cleaned with Virusolve (Amity International). The test session videos were scored and analyzed with EthoVision-XT software (Noldus Information Technology).

### 4.6 Conditioned place preference test

The CPP test was performed with the UVB or control-treated mice when they were under the 22-h food deprivation. The CPP conditioning chambers were translucent acrylic boxes (33 × 18 × 16 cm^3^) with removable clear plastic tops and retractable doors. The arena had one center zone and two side zones; the first with the reward food which was the non-textured zone, and the second without food, which was the black and white textured zone. Both the zones had a cap of a 50-mL tube which was or was not filled with the food. The experimental room was lit by normal white light (300 Lux), and behavior-related parameters were recorded by video for 15 min from the top and analyzed using EthoVision-XT software (Noldus Information Technology). Between each trial, the arena was cleaned with Virusolve (Amity International). Pellets consumed, distance traveled, and total activity were measured.

The CPP test was performed in three phases: (1) habituation (Day 0), (2) training (Days 1–8), and (3) test (Day 9). On day 0, no food was placed in either chamber. The mouse was placed in the center of the arena, the doors were retracted, and the mouse was allowed to move freely in the arena for 15 min.

During training, reward food pellets (~ 100 pellets per session) were introduced in zone 1 (the light zone), and there were no pellets in the textured zone 2 (the dark zone). Each mouse was placed with access to the reward food zone 1 (light zone) or in textured zone 2 (dark zone) on alternate days. The doors were closed to restrict access only to the zone in which the mouse was placed. The number of pellets consumed during the 15 min in which the mouse was in the zone were calculated.

On Day 9, mice were introduced into the CPP arena with doors retracted to allow free movement in the arena. No pellets were supplied. Mice were allowed to move freely for 15 min, and the duration of time spent in each zone was measured.

### 4.7 Elevated-plus maze test

The test was performed using the setup described previously (Parikh et al., 2021). In short, the test was performed using EPM maze with a height of 90 cm lit by normal white light (300 Lux) in the experiment room. The maze included two opposing closed arms (arm lengths 40 cm, arm widths 5 cm, wall height 15 cm), and an open 5 × 5 cm square in the center. The experimental animals were placed in the center facing an open arm, center time spent, and velocity behavior-related parameters were measured for 5 min using the camera from the top and analyzed using EthoVision-XT software (Noldus Information Technology). Between each trial, the maze was cleaned with Virusolve® + (Amity International).

### 4.8 Statistics

All the experimental data are shown as means and standard errors of the mean. We used a random experimental design, Student’s *t*-tests (two-tailed) for two-group comparisons, and ANOVAs for multiple group comparisons followed by Tukey’s multi-comparison tests. At relevant places the *t*-statistics (with df) and *F*-statistics (*F*, DFn, DFd) is mentioned in the text but all the related statistical details appear in Supplementary Tables S1–S3.

## Data availability statement

The original contributions presented in the study are included in the article/Supplementary material, further inquiries can be directed to the corresponding authors.

## Ethics statement

The animal study was approved by Tel Aviv University Institutional Animal Care and Use Committee. The study was conducted in accordance with the local legislation and institutional requirements.

## Author contributions

SP: Formal analysis, Investigation, Writing – review & editing, Conceptualization, Data curation, Methodology, Writing – original draft. RP: Formal analysis, Writing – review & editing, Investigation. AW: Formal analysis, Methodology, Writing – review & editing. LB: Conceptualization, Investigation, Methodology, Supervision, Writing – original draft, Writing – review & editing. CL: Conceptualization, Data curation, Funding acquisition, Investigation, Methodology, Resources, Supervision, Writing – original draft, Writing – review & editing. MH: Formal analysis, Statistics.
